# Epicardial electroanatomical mapping, radiofrequency ablation, and lesion imaging in the porcine left ventricle under real-time magnetic resonance imaging guidance—an *in vivo* feasibility study

**DOI:** 10.1093/europace/eux341

**Published:** 2017-12-26

**Authors:** Rahul K Mukherjee, Sébastien Roujol, Henry Chubb, James Harrison, Steven Williams, John Whitaker, Louisa O’Neill, John Silberbauer, Radhouene Neji, Rainer Schneider, Thomas Pohl, Tom Lloyd, Mark O’Neill, Reza Razavi

**Affiliations:** 1 Division of Imaging Sciences and Biomedical Engineering, King's College London, 4th Floor, North Wing, St Thomas' Hospital, Westminster Bridge Road, London, UK; 2 Department of Cardiology, Brighton and Sussex University Hospital NHS Trust, Eastern Road, Brighton, UK; 3 Siemens Healthcare, Sir William Siemens Square, Frimley, Camberley, UK; 4 Siemens Healthcare GmbH Erlangen, Germany; 5 Imricor Medical Systems, 400 Gateway Blvd, Burnsville, MN, USA

**Keywords:** Ventricular tachycardia, Magnetic resonance thermometry, Catheter ablation, Epicardial, Real time Magnetic resonance imaging, T1 mapping

## Abstract

**Aims:**

Magnetic resonance imaging (MRI) is the gold standard for defining myocardial substrate in 3D and can be used to guide ventricular tachycardia ablation. We describe the feasibility of using a prototype magnetic resonance-guided electrophysiology (MR-EP) system in a pre-clinical model to perform real-time MRI-guided epicardial mapping, ablation, and lesion imaging with active catheter tracking.

**Methods and results:**

Experiments were performed *in vivo* in pigs (*n* = 6) using an MR-EP guidance system research prototype (Siemens Healthcare) with an irrigated ablation catheter (Vision-MR, Imricor) and a dedicated electrophysiology recording system (Advantage-MR, Imricor). Following epicardial access, local activation and voltage maps were acquired, and targeted radiofrequency (RF) ablation lesions were delivered. Ablation lesions were visualized in real time during RF delivery using MR-thermometry and dosimetry. Hyper-acute and acute assessment of ablation lesions was also performed using native T1 mapping and late-gadolinium enhancement (LGE), respectively. High-quality epicardial bipolar electrograms were recorded with a signal-to-noise ratio of greater than 10:1 for a signal of 1.5 mV. During epicardial ablation, localized temperature elevation could be visualized with a maximum temperature rise of 35 °C within 2 mm of the catheter tip relative to remote myocardium. Decreased native T1 times were observed (882 ± 107 ms) in the lesion core 3–5 min after lesion delivery and relative location of lesions matched well to LGE. There was a good correlation between ablation lesion site on the iCMR platform and autopsy.

**Conclusion:**

The MR-EP system was able to successfully acquire epicardial voltage and activation maps in swine, deliver, and visualize ablation lesions, demonstrating feasibility for intraprocedural guidance and real-time assessment of ablation injury.

**What’s new?**

- Epicardial activation and voltage maps were acquired under real-time magnetic resonance imaging (MRI) guidance *in vivo* on a large animal model solely using active catheter tracking. High-quality intracardiac electrograms were recorded from the epicardium during MR scanning with a signal-to-noise ratio >10:1 for a signal of 1.5 mV.
- Quantitative thermal dose mapping during real-time MR-thermometry allowed visualization and measurement of lesion dimensions during epicardial ablation.
- Myocardial native T1 mapping measurements demonstrated decreased T1 times in the ablation lesion core and elevated T1 times in surrounding regions of ablated tissue (relative to non-ablated tissue) within 3–5 min following delivery of epicardial radiofrequency ablation.

## Introduction

Radiofrequency (RF) catheter ablation reduces recurrences of ventricular tachycardia (VT) and appropriate implantable cardioverter defibrillator (ICD) therapies in patients with ischaemic^1^^,^^2^ and non-ischaemic cardiomyopathy.^3^ However, catheter ablation is still limited by relatively low long-term success rates. Catheter ablation of VT has evolved from mapping and ablation of electrophysiologically defined re-entrant circuits to the identification and targeting of abnormal myocardial substrate (areas of scar and surrounding border zone containing a mixture of healthy and scar tissue). Although electrical surrogates of arrhythmogenic myocardium are assessed by contact electroanatomical mapping (EAM) of the endocardium and epicardium, there are several limitations to this approach. Electroanatomical mapping cannot interrogate the complex 3D anatomy of scar; the spatial resolution of voltage-based mapping is limited by multiple factors including electrode size, orientation, contact, and number of points collected; electrical criteria for scar annotation may not be universally applicable to pathological scar.^4^ In patients with non-ischaemic cardiomyopathy who may have epicardial or intramural scar, the limitation of EAM to delineate fully the 3D geometry of scar has been well reported.^5^

Magnetic resonance imaging (MRI) using late-gadolinium enhancement (LGE) is the gold standard for non-invasive imaging of scar/fibrosis.^6^ Late-gadolinium enhancement can be used for defining myocardial substrate in 3D and potentially identify channels of slow conduction prior to VT ablation.^7^ These can be channels between areas of scar core or scar border zone (described as grey zone on LGE-MRI), where there is a mixture of normal and scarred myocardium and associated with slow conduction.^8^ In patients with non-ischaemic cardiomyopathy with epicardial substrate, where endocardial-only ablation is less likely to be successful, LGE-MRI has been correlated to areas of low epicardial voltage and used to guide epicardial ablation.^9^

Real-time MRI-guided EAM and ablation offers an attractive strategy to improve the precision of substrate identification, provide intraprocedural guidance, and facilitate assessment of ablation lesion formation. Previous work in this area has focused on characterizing correlations between MRI and EAM,^10^ device tracking (using passive and active catheter tracking),^11^ assessment of electrogram fidelity in the magnetic resonance (MR) environment,^12^ and lesion evaluation.^13^ To date, however, there are no studies evaluating epicardial mapping and ablation under real-time MRI guidance.

In this study, we report the feasibility of performing epicardial mapping and ablation under real-time MRI guidance using active catheter tracking in a porcine model. We also report on the use of real-time lesion assessment with MR-thermometry for epicardial ablation, hyper-acute lesion imaging with native T1 mapping and acute lesion assessment with LGE.

## Methods

### Animal model and preparation

Animal studies complied with the French law on animal experiments and were performed at the Institut de Chirurgie Guidée par l’Image (IHU), Strasbourg, France. Six female Landrace pigs (46.7 ± 10 kg) were pre-sedated with a combination of intramuscular tiletamine and zolazepam. General anaesthesia was induced and maintained with inhaled 1–5% isoflurane, and the animals were intubated and mechanically ventilated (20–25 breaths/min). Vascular access (7 Fr introducer sheath) for invasive monitoring, and administration of medications was obtained through the right and left femoral arteries and femoral vein using ultrasound guidance. Epicardial access was gained using an anterior percutaneous subxiphoidal puncture under X-ray guidance in a fluoroscopy suite. A custom MR-compatible 10 Fr deflectable sheath (Imricor, Burnsville, MN, USA) was placed within the pericardial space for epicardial access. An MR-compatible steerable catheter of 9 Fr with open irrigation (Vision-MR, Imricor, Burnsville, MN, USA) was manoeuvred into the epicardial space. The catheters have been described in detail previously.^14^ A custom MR-EP recording system (Advantage-MR) consisting of a digital amplifier and stimulator, patient information module, and a host workstation was used to record, display, and analyse intracardiac signals; to act as a programmable stimulator; and to deliver ablation energy as previously described.^14^ All animals underwent invasive blood pressure and surface electrocardiogram (ECG) monitoring (Invivo Medical, Gainesville, FL, USA) throughout the study. Anticoagulation was maintained with an intravenous bolus of 70 units/kg of heparin. A continuous infusion of intravenous lidocaine (12 mL/h) and amiodarone (20 μg/kg/min) was administered prior to mapping studies and ablation. A bolus of intravenous amiodarone (150 mg in 60 mls dextrose) was administered as a slow push prior to each ablation lesion delivered. Animals were transferred to an MRI suite following epicardial access for mapping and ablation studies.

All MRI scans described below were performed on a 1.5 T MR scanner (MAGNETOM, Aera, Siemens Healthcare, Erlangen, Germany).

### Active catheter tracking

To project the location of the ablation catheter within the context of the cardiac chambers, active catheter tracking using a dedicated MRI tracking sequence, detected by microcoils within the catheter was used. A 3D bSSFP whole heart volume was acquired at the start of the procedure (described below), and manual chamber segmentations were performed to provide an interface that closely mimicked that of a clinical EAM-style system.

To perform active catheter tracking during MRI scans, the *X*, *Y*, *Z* coordinates of the catheter microcoils were determined using a custom active tracking sequence/module, which was optionally interleaved with a fast balanced steady-state free precession (bSSFP) imaging sequence automatically following the current catheter position. The active tracking sequence comprised three non-selective projection acquisitions along the respective axis. A dynamic imaging coil detuning approach and pre-spoiler were applied to avoid potential background noise, i.e. coil coupling and residual signal effects. Based on the acquired projections, the corresponding signal peaks were detected with a dynamic template-matching algorithm, which used the initial projections to calculate a template per coil and axis. The template was continuously updated with each new projection fulfilling a minimal peak-to-noise ratio to adapt to the changing shape of the projections while manoeuvring the catheter. The detected positions were fed back to both the iCMR platform (Siemens Healthcare) and the MRI scanner to update the rendered catheter position/orientation and imaging plane location, respectively. The tracking module was further optimized to run with an ambient acoustic noise and had been found to perform robustly with an open MR cabin door, in the presence of other electrical equipment (e.g. ventilator, ECG monitoring, etc.) and during RF ablation.

### 3D whole heart sequence

A 3D, ECG-triggered whole-heart bSSFP acquisition with a 1D diaphragmatic navigator (5 mm gating window) was performed with the following parameters: transverse slice orientation, AP phase encoding, 1.25 × 1.25 mm^2^ in-plane resolution, 256 × 256 in-plane matrix size, 2.5 mm acquired slice thickness reconstructed to 1.25 mm, typically around 100 slices to cover the whole heart, TR/TE = 3.7/1.64 ms, flip angle = 90°, readout bandwidth (per pixel) = 592 Hz, fat suppression, and GRAPPA acceleration factor = 2. The trigger delay was set so that the acquisition was performed during mid-diastole, and the acquisition window was set to correspond to the length of the diastolic period (typically ∼110 ms, corresponding to 30 k-space lines acquired per heartbeat). Following acquisition, the left ventricle, right ventricle, left atrium, right atrium, and aorta were manually segmented on each animal using an image processing platform based on a version of Medical Imaging Interaction Toolkit (MITK, Heidelberg, Germany) and saved as Stereolithography (.STL) files. Segmentations were loaded on the iCMR application (Siemens Healthcare) to create a road map for mapping and ablation studies.

### Epicardial mapping and ablation

Real-time intracardiac electrograms were recorded from the epicardium and displayed using the Advantage-MR EP Recorder/Stimulator System (Imricor Medical Systems, Burnsville, MN, USA; see Supplementary material online, *Figure S1*). Each intracardiac electrogram (EGM) signal was displayed at 1 mV/cm to measure the peak-to-peak signal amplitude and at 0.1 mV/cm to measure the peak-to-peak noise amplitude. Epicardial voltage maps were displayed with threshold of <1.5 mV. Once epicardial EGM fidelity in the MR environment was demonstrated as feasible, we committed to delivering RF ablations in remaining animals. Ablation of the left ventricle was performed by delivering RF energy (40–60 W, irrigation rate 17 mL/min, and 50–60 s duration). Impedance change and catheter tip temperature were continuously assessed. Ten discrete ablation lesions were delivered in four animals with lesion position automatically recorded in 3D space and annotated upon the 3D rendering of each heart on the iCMR application. The aim was to create transmural lesions during each discrete ablation. Using measurements on gross macroscopic examination as the gold standard, conformational accuracy of lesion annotation on the iCMR application was assessed using the angle of intersection between points on two ablation lines measured to the nearest degree. Spatial accuracy was referenced to the LV apex and was measured to the nearest millimetre.

### Cardiovascular magnetic resonance-thermometry

Real-time lesion imaging could offer an attractive means to titrate energy delivery which could potentially decrease overall procedural time, increase efficacy, and reduce procedural risk during MR-guided EP procedures. In this study, we employed cardiovascular magnetic resonance (CMR)-thermometry using the proton resonance frequency shift (PRFS) technique, which is sensitive to temperature changes in real time and has been most widely studied in non-cardiac ablation.^15^

To perform CMR-thermometry, an ECG-triggered multi-slice single-shot echo planar imaging (EPI) sequence was used with spoiled gradient echo (TR/TE/α = 50 ms/17 ms/60°, FOV = 180 × 180 mm^2^, voxel size = 1.6 × 1.6 mm^2^, slice thickness = 5 mm, slice number = 4, bandwidth = 1565 Hz/Px, GRAPPA factor = 2, partial Fourier = 0.75, orientation—short axis). Saturation slabs were used for reduced field of view imaging and inflow saturation to reduce blood signal.^16^ Reconstruction of temperature maps was performed offline using a customized multi-baseline approach extending the method previously proposed^15^ as follows: a look up table of co-registered CMR-phase images was initially created and the CMR-thermometry sequence was run for 20 heartbeats before each ablation. Images from the first heartbeat served as the reference image. Non-rigid motion was then estimated between the reference and each subsequent magnitude image using an optical flow technique. Phase images were then registered to the reference position using the estimated motion fields. The registration was performed on the complex data instead of the phase images to prevent artefacts due to phase wrap. During ablation, each new phase image was registered to the reference, position as described above. The phase image from the lookup table that best matched the current registered phase image (i.e. minimal mean square error between pairwise unwrapped phase images) was then selected and used for temperature estimation.

### Cardiovascular magnetic resonance dosimetry

During RF ablation, the ideal lesion will likely be transmural with no extracardiac extension but create permanent tissue destruction to prevent the recurrence of arrhythmia. There is only a small body of literature, however, demonstrating direct assessment of lesion formation during RF delivery. Cardiovascular magnetic resonance-dosimetry derived from MR-thermometry is a technique that can be used to provide a real-time accumulated thermal dose (TD) during RF energy delivery at the intended ablation point. Thermal dose mapping was calculated on a pixel-wise basis as follows as previously described^13^:

$$\text{TD}=\{\begin{array}{c} \int_{0}^{t}\begin{array}{cc} 2\left(T(t)-43\right)dt & \text{if }T(t) \end{array}>43\;{}^{\circ}\text{C}\\ \int_{0}^{t}\begin{array}{cc} 4\left(T(t)-43\right)dt & \text{if }T(t) \end{array}<43\;{}^{\circ}\text{C} \end{array},$$

where T(t)represents absolute tissue temperature. Baseline temperature was approximated to 39 °C. The TD was considered as lethal when exceeding the equivalent TD at constant heating of 43 °C for 240 s. To correct for TD error induced by noise on temperature estimates, TD maps were corrected on a pixel-wise basis as follows:

$${\text{TD}}_{\text{corr}}=\text{TD}\cdot e^{-0.5\left(\ln\left(2\right)\cdot \sigma_{\text{T}}\right)},$$

where TD_corr_ is the corrected TD and $\sigma_{T}$is the temporal standard deviation of temperature estimates at baseline (measured from the first 20 measurements following the training thermometry step and before the heating process). Width and depth of lesion estimated from TD mapping were measured from the middle slice containing the largest lethal dose area.

### Native T1 mapping

The longitudinal relaxation time constant (T1) of the myocardium is known to be altered following RF ablation.^17^ Post-procedural MRI using native T1 mapping therefore presents an opportunity for the assessment of ablation injury. The modified Look-Locker (MOLLI) sequence was used for native myocardial T1 mapping using a 5-(3)-3 scheme and a bSSFP readout (TR/TE/α = 2.5 ms/1.2 ms/35°, FOV = 360 × 307 mm^2^, voxel size = 1.4 × 2.1 mm^2^, slice thickness = 8 mm, bandwidth = 1085 Hz/Px, GRAPPA factor = 2, partial Fourier = 0.87). Average T1 times were measured in regions of interest (ROIs) manually drawn on the MOLLI scans in areas of ablated tissue. Similarly, reference T1 measurements were calculated for the remote LV myocardium in non-ablated tissue by drawing ROIs on the anteroseptal wall. Approximate lesion location identified on T1 mapping was correlated with lesion location identified on LGE imaging.

### Late-gadolinium enhancement imaging

High-resolution 3D LGE imaging was performed using a free-breathing navigator-gated inversion recovery gradient echo (GRE) sequence (TR/TE/α = 5.45 ms/1.8 ms/10°, FOV = 339 × 264 × 100 mm^3^, voxel size = 1.3 × 1.3 × 4 mm^3^, bandwidth = 360 Hz/Px, GRAPPA factor = 2, 2RR acquisition). Magnevist (gadopentetate dimeglumine) (Bayer, Germany) 0.1 mmol/kg was administered following completion of all ablation lesions and the imaging sequence run 10–15 min following contrast administration.

### Histological analysis

Following euthanasia (performed immediately after completion of mapping and ablation studies), hearts were explanted, photographed, and fixed in 10% formalin. The ablation lines and surrounding tissue were excised *en bloc* and cut into sections perpendicular to the ablation line. Lesions were photographed, while depth and width was measured with a ruler and dimensions correlated to those observed on 2D TD maps. Each cross-section was dehydrated, embedded in paraffin, sectioned (6 μm sections), and then stained with haematoxylin and eosin.

### Statistical analysis

Data analysis was performed using GraphPad Prism 7 (GraphPad Prism, San Diego, CA, USA). Data were displayed as mean ±standard deviation. Differences between groups were analysed using independent Student’s *t*-test for unpaired data. The value of *P* < 0.05 was considered statistically significant.

### Experimental ethics and reporting

The experimental protocol in the animal model was approved by the local ethics committee and was compliant with the Guiding Principles for the Use and Care of Animals published by the National Institutes of Health (NIH Publication no. 85-23, Revised 1996).

## Results

**Figure 1:**
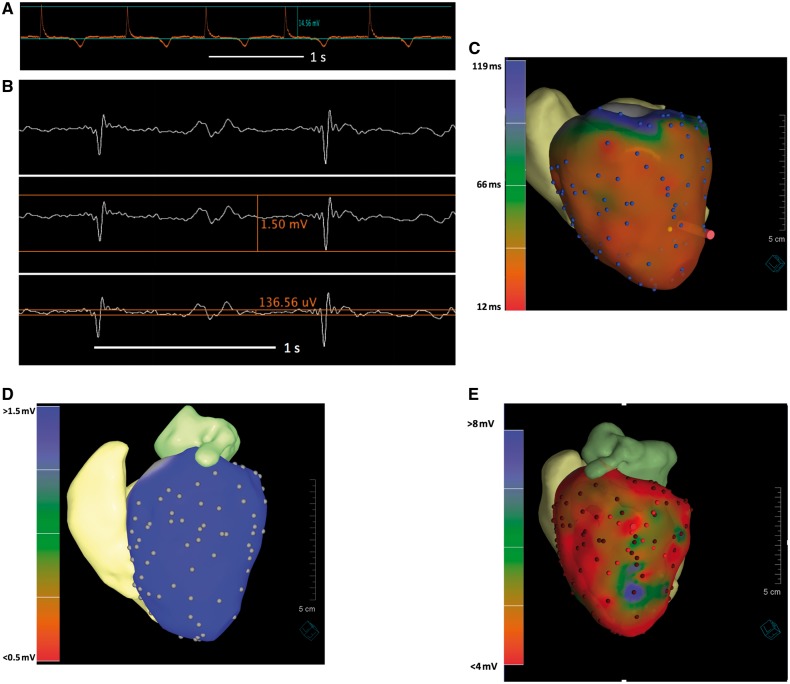
Intracardiac EGM signal quality, epicardial activation, and voltage maps. (*A*) Representative intracardiac EGM from the epicardium showing a peak amplitude of 14.56 mV (0.97 mV noise level)—sweep speed 100 mm/s. (*B*) For a low voltage 1.5 mV signal, a noise level of 0.136 mV is present giving a >10:1 signal-to-noise ratio. (*C*) Epicardial activation map in swine in sinus rhythm generated using active catheter tracking only in the MR environment. (*D*) Epicardial voltage map with threshold set between 0.5 mV and 1.5 mV. (*E*) Epicardial voltage map with a threshold set between 4.0 mV and 8.0 mV. 3D shells shown were created following segmentation of the whole heart sequence from MRI and imported into the iCMR application. Activation and voltage map data were superimposed onto the anatomical shells.

Epicardial mapping was performed solely under real-time MRI guidance with active catheter tracking in two animals. The total procedural time to complete epicardial mapping was 32 min (101 points) in the first animal (*Figure **1*) and 20 min (56 points) in the second animal. High-quality intracardiac electrograms were recorded in the MR environment to build the EAMs Representative results of the electrogram signal quality are shown in *Figure **1*—a peak-to-peak amplitude of 14.56 mV is seen for a typical epicardial signal. A mean signal-to-noise ratio of 32.8 (±14.9) was calculated for epicardial points taken. For a signal (low voltage) of 1.5 mV, a noise level of 0.14 mV was seen with a better than 10:1 signal-to-noise ratio observed (*Figure **1*).

A further four animals had 10 discrete RF ablations delivered on the epicardium (40–60 W for 50–60 s duration) with catheter navigation performed solely with active tracking. Ablation lesions annotated on the anatomical shells (iCMR application, Siemens Healthcare) corresponded well with post-ablation lesion location on autopsy (*r* = 0.97) with a mean spatial accuracy of 2.2 mm (±1.5 mm) and a mean conformational accuracy of 4.4° (±1.8°) (see Supplementary material online).

### Real-time lesion visualization

**Figure 2:**
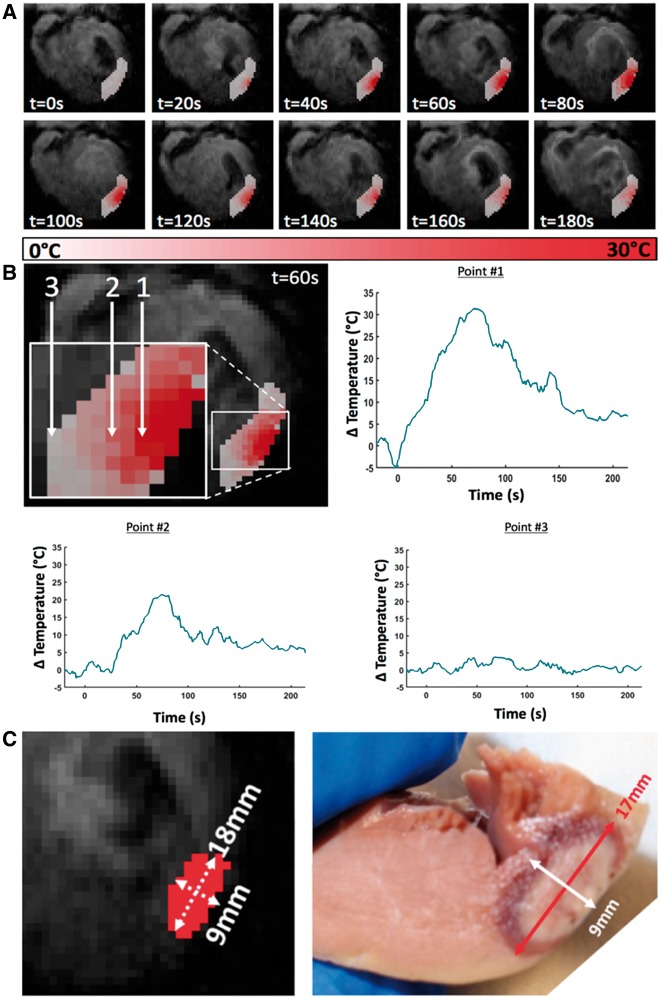
*In vivo* Δtemperature maps (*A*) shown at different time points relative to the start of the RF heating. Localized temperature elevation can be clearly visualized on the epicardial side of the left ventricle. Temporal profiles (*B*) obtained using CMR-thermometry during epicardial ablation in swine. A maximum temperature elevation of 35 °C was observed in a pixel within 2 mm of the catheter tip—Point 1 (relative to a more remote pixel in myocardium >8 mm away from the catheter tip—Point 3). No significant temperature elevation was observed in a pixel located in a remote area. 2D lesion dimensions measured using MR-dosimetry (*C*) correlate well with measurements on gross macroscopy with mild overestimation of lesion width.

MR-thermometry and dosimetry was able to demonstrate a localized temperature elevation near the catheter tip during delivery of epicardial RF ablation. A maximum temperature difference of 35 °C (relative to pixel in area of more remote septum) was observed within 2 mm of the irrigated catheter tip, while a lower temperature difference was observed further away from the catheter tip in the myocardium and no temperature change in a more remote area of myocardium >8 mm away from the catheter tip (*Figure **2*).

### Hyper-acute and acute lesion visualization

**Figure 3:**
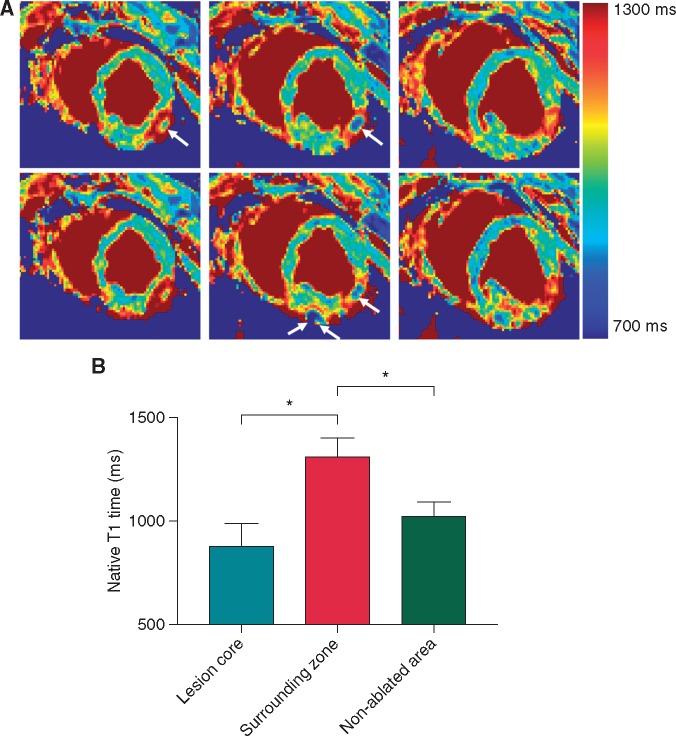
Native T1 maps at six slices (apical to basal) 3–5 min following delivery of an ablation lesion (*A*) decreased T1 times were seen in the lesion core whilst elevated T1 times were seen in the surrounding tissue relative to non-ablated myocardium (ablation lesion core in white arrows). Mean native T1 time in the lesion core was 882 ms (±107 ms), in surrounding tissue was 1316 ms (±85 ms), and 1028 ms (±64 ms) in remote, non-ablated tissue (*B*); *n* = 10 lesions; *P < 0.05.

Mean native T1 time in the core of ablation lesions was decreased (relative to non-ablated myocardium) at 882 ms (±107 ms) while in surrounding tissue, mean native T1 times were elevated at 1316 ms (±85 ms). In remote, non-ablated tissue, mean native T1 times were 1028 ms (±64 ms) (*Figure **3*). Areas of elevated native T1 times matched well with areas of hyper-enhancement on LGE images and to lesion location on pathological examination (see Supplementary material online).

### Gross macroscopy and histology

On gross macroscopy, a zone of pallor could be clearly delineated corresponding to the ablation lesion core with a surrounding haemorrhagic zone (see Supplementary material online). Lesion transmurality of >75% was seen in 9 of 10 ablation lesions. For measurement of lesion dimensions, the ablation lesion was defined as both the zone of pallor and the surrounding oedematous zone. *Figure **2* illustrates representative short-axis images on MR-dosimetry to measure lesion dimensions with corresponding lesion delineation on gross macroscopy. Lesion dimensions measured using MR-dosimetry matched well to dimensions on gross macroscopy with mild overestimation (*Figure **2*). Microscopic histology of acute ablation injury on haematoxylin and eosin staining demonstrated partial loss of membrane borders, nuclear elongation, and interstitial oedema (see Supplementary material online).

## Discussion

This study demonstrates the feasibility of using real-time MRI guidance to perform epicardial activation/voltage mapping and RF ablation using active catheter tracking in a porcine model. Estimated lesion dimensions by real-time MR-dosimetry during epicardial RF ablation corresponded to gross macroscopy. Native T1 time decreased in the lesion core and was elevated in the surrounding areas of ablated tissue within minutes of delivering epicardial RF ablation.

### Demonstration of intracardiac EGM fidelity

The accurate detection of cardiac electrical activity is difficult in the MR environment in part due to the presence of time variable gradient fields and magnetohydrodynamic (MHD) effects. Intracardiac electrograms may be high-pass and low-pass filtered, often requiring a further notch filter to reduce gradient signal-induced noise of intracardiac electrograms. Elbes *et al.*^18^ found that the ‘noise’ amplitude inside the MR environment varied according to the type of sequence, slice orientation, gradient amplitude, and switching times with a peak-to-peak noise amplitude ranging from 0.025 to 0.700 mV. Nazarian *et al.*^12^ reported a filtered ventricular electrogram SNR of 71.6 compared with an unfiltered SNR of 3.7 in the endocardial RV apex using active catheter tracking in mongrel dogs.

To the best of our knowledge, there are no studies investigating intracardiac EGM fidelity during epicardial mapping under real-time MRI guidance solely with active catheter tracking. Epicardial substrate mapping is arguably more challenging than endocardial mapping due to the presence of epicardial fat and low-voltage areas along the course of large coronary arteries and the atrio-ventricular (AV) groove, affecting the reliability of electrograms recorded.^9^ Our study demonstrates that it is feasible to detect a good signal quality during epicardial mapping in the MR environment with an acceptable noise level and a >10:1 signal-to-noise ratio for low-voltage signals.

### Demonstration of MR-EP tracking and therapy accuracy

The use of active catheter tracking with a dedicated tracking sequence, detected by microcoils in a MR-compatible catheter has been employed previously.^19^ The microcoil method is advantageous due to its ability to track multiple coils along the body of the catheter, a faster rate of tracking and choice of guidance using acquired road maps or real-time imaging.^19^ Grothoff *et al.*^14^ reported the use of active catheter tracking to guide intubation of the coronary sinus, trans-septal puncture, activation mapping of the left atrium, and ablation of the AV node in pigs. Catheter position was confirmed by passive real-time imaging. We have previously demonstrated the feasibility of real-time MRI-guided catheter ablation in patients with typical atrial flutter using active catheter tracking.^19^ Using a combination of active tracking and catheter visualization with real-time MR imaging, Hilbert *et al.*^11^ also performed cavotricuspid isthmus ablation in six patients with a mean procedural time of 109± 58 min. Complete isthmus block was achieved in three of six patients without additional fluoroscopy. There are little data, however, demonstrating the accuracy of epicardial RF ablation in a real-time MR environment, even in pre-clinical models.

In this study, following demonstration of the feasibility of epicardial mapping and ablation using active catheter tracking, progress has been made on a complete MR-based solution for VT substrate modification.

### Real-time lesion visualization

There has been significant interest in the development of MR tools to assess ablation lesions in real time. Direct monitoring of tissue temperature using MR-thermometry and dosimetry offers an exciting means of exploiting acute physiological changes and could potentially be used to improve the safety and efficacy of catheter ablation. Kolandaivelu *et al.*^20^ first described the use of MR-thermometry using the proton resonance frequency (PRF) shift technique to quantify tissue temperature changes that lead to ablation lesion formation. In mongrel dogs, the maximum ablation lesion extent during endocardial RF ablation on MR-thermometry corresponded well to the lesion location, depth on LGE-MRI, and pathological examination.^20^ Lesion transmurality with MR-thermography was within 20% of that measured by pathology and LGE-MRI. Recently, in an ovine model, Toupin *et al.*^13^ demonstrated that endocardial ablation lesion dimensions on TD images correlated well with 3D T1-weighted images acquired immediately after ablation and during gross pathological examination. The precision of lesion extent in the myocardium was in the region of 1 mm—potentially offering a useful MR tool in real time to guide the safety and efficacy of RF ablation. Our report extends the use of this technique to provide an assessment of epicardial lesion extent during RF ablation. Further work is still needed, however, to assess how real-time lesion imaging relates to chronic transmural lesions—a porcine recovery model of ventricular ablation could be used to confirm long-term efficacy in future studies. In our study, the TD model was used for the prediction of ablation lesions. Other approaches have also been proposed such as using the maximum temperature peak for each voxel.^13^^,^^20^ Further work is needed to establish the most accurate model for the prediction of permanent ablation lesions.

### Hyper-acute and acute lesion visualization

Our data indicate that within minutes of RF ablation, there is a decrease in native T1 time in the lesion core and an overall localized elevation in native T1 times at the surrounding areas of ablation lesions compared with areas of non-ablated tissue. Although there are few studies investigating the use of native T1 mapping to visualize the effect of RF ablation within this time frame, it has been suggested that catheter ablation induces changes in native tissue T1 time.^17^ Following catheter ablation, there may be several types of tissue injury present within the lesion core and surrounding areas including necrotic tissue, haemorrhage, oedema, and thrombus. Recent work suggests that *in vivo* MOLLI measurements show different T1 times at the lesion core (decreased T1) and lesion periphery (increased T1) when imaging is performed within 2 h of ablation in a porcine model.^17^ Due to the different types of tissue injury involved, it may be inappropriate to evaluate ablation lesions using an overall mean native T1 time in ablated zones. Further, native T1 mapping may also be inappropriate to assess ablation lesions in patients with non-ischaemic cardiomyopathy where differences in T1 times may exist between relatively healthy and diseased segments of myocardium due to the underlying cardiomyopathy making interpretation difficult following ablation. Further work is required in this area to assess the effects of RF ablation on native T1 times at different time-points after ablation with histopathological validation.

Of the different approaches described in this study, to assess acute ablation injury (thermometry/dosimetry, native T1 mapping, and LGE), thermometry remains the only technique with the ability to assess lesions in real-time. To facilitate the real-time MR-guided ablation, thermometry would be our preferred approach for lesion assessment. However, there are several limitations to the technique in its current form that need to be overcome (described below).

### Limitations

There are several important limitations to this work. The purpose of this preliminary study was to demonstrate the feasibility of performing epicardial mapping and ablation under real-time MRI guidance with active catheter tracking in a pre-clinical setting. As such, the epicardial electroanatomical maps generated were of a low resolution (101 and 56 points) and only obtained in normal hearts. Further work is required to assess the procedural time of high-resolution epicardial activation and voltage maps under real-time MRI guidance, ideally in an animal model with areas of scar and low voltage. Following ventricular RF ablation, large animal models frequently become tachycardic and/or develop ventricular arrhythmias that may affect the quality of the myocardium, blood, and phase signal during MR-thermometry. MR-thermometry in the presence of irregular heart rhythm was not evaluated in this study, and its feasibility remains to be established. Further refinements in the thermometry technique may be needed to overcome these limitations. We did not evaluate chronic ablation injury in this model; therefore, further work is needed to evaluate whether TD achieved during acute lesions corresponds to permanent lesion formation in a recovery model. The sensitivity and specificity of MR-thermometry for lesion monitoring during ablation was not assessed, while the limited number of lesions delivered precluded our ability to assess whether the extent of temperature rise could be predictive of lesion transmurality—these areas could be an important focus of future work. The thermometry data required post-processing offline to generate the data on lesion dimensions. For the data to be useful to the electrophysiologist, a real-time pipeline with visualization of the TD onto an anatomical shell and demonstration of lesion dimension needs to be developed to guide catheter ablation although this was beyond the scope of this preliminary work. To facilitate VT ablation under MRI guidance in patients, there are still significant hurdles to overcome such as the availability of MRI-conditional defibrillation systems and MR-compatible 12-lead ECG systems. Patients undergoing VT ablation frequently require defibrillation—the time delay associated with removing a patient from the scanner bore before delivery of cardiopulmonary resuscitation and defibrillation could potentially increase the risk of mortality. Although there are prototype MR-compatible defibrillation systems currently undergoing testing, until there are robust systems that are shown to be successfully able to defibrillate patients without increasing the time needed for defibrillation, the uptake of higher risk MR-guided interventions is likely to be limited.

## Conclusion

This study demonstrates the feasibility of epicardial electroanatomical mapping and ablation under real-time MRI guidance with active catheter tracking in the porcine left ventricle. MR-thermometry and dosimetry enabled the real-time visualization of ablation injury and measurement of lesion dimensions whilst changes in native T1 times were observed within minutes of lesion delivery.

## Supplementary Material

Supplementary Data

## References

[1] Kuck K-H, Schaumann A, Eckardt L, Willems S, Ventura R, Delacrétaz E et al Catheter ablation of stable ventricular tachycardia before defibrillator implantation in patients with coronary heart disease (VTACH): a multi-centre randomised controlled trial. Lancet 2010;375:31–40. 20109864 10.1016/S0140-6736(09)61755-4

[2] Reddy VY, Reynolds MR, Neuzil P, Richardson AW, Taborsky M, Jongnarangsin K et al Prophylactic catheter ablation for the prevention of defibrillator therapy. N Engl J Med 2007;357:2657–65. 18160685 10.1056/NEJMoa065457 PMC2390777

[3] Muser D,, Santangeli P,, Castro SA,, Pathak RK,, Liang JJ,, Hayashi T et al Long-term outcome after catheter ablation of ventricular tachycardia in patients with non-ischaemic dilated cardiomyopathy. Circ Arrhythm Electrophysiol 2016;9:e004328. 27733494 10.1161/CIRCEP.116.004328

[4] Ashikaga H,, Sasano T,, Dong J,, Zviman MM,, Evers R,, Hopenfeld B et al Magnetic resonance-based anatomical analysis of scar-related ventricular tachycardia: implications for catheter ablation. Circ Res 2007;101:939–47. 17916777 10.1161/CIRCRESAHA.107.158980 PMC2842927

[5] Piers SR, Zeppenfeld K. Imaging-guided ventricular tachycardia ablation. Arrhyth Electrophysiol Rev 2013;2:128–34. 10.15420/aer.2013.2.2.128 PMC4711544 26835054

[6] Kim RJ, Wu E, Rafael A, Chen E-L, Parker MA, Simonetti O et al The use of contrast-enhanced magnetic resonance imaging to identify reversible myocardial dysfunction. N Engl J Med 2000;343:1445–53. 11078769 10.1056/NEJM200011163432003

[7] Andreu D, Ortiz-Pérez JT, Fernández-Armenta J, Guiu E, Acosta J, Prat-González S et al 3D delayed-enhanced magnetic resonance sequences improve conducting channel delineation prior to ventricular tachycardia ablation. Europace 2015;17:938–45. 25616406 10.1093/europace/euu310

[8] Arenal Á, Pérez-David E, Ávila P, Fernández-Portales J, Crisóstomo V, Báez C et al Non-invasive identification of epicardial ventricular tachycardia substrate by magnetic-resonance based signal intensity mapping. Heart Rhythm 2014;11:1456–64. 24747421 10.1016/j.hrthm.2014.04.022

[9] Reithmann C, Herkommer B, Fiek M. Epicardial ventricular tachycardia substrate visualised by magnetic resonance imaging: need for a transpericardial ablation approach? Clin Res Cardiol 2016;105:827–37. 27294860 10.1007/s00392-016-0990-0

[10] Oduneye SO, Pop M, Shurrab M, Biswas L, Ramanan V, Barry J et al Distribution of abnormal potentials in chronic myocardial infarction using a real-time magnetic resonance guided electrophysiology system. J Cardiovasc Magn Reson 2015;17:17–27. 25890360 10.1186/s12968-015-0133-1 PMC4392456

[11] Hilbert S, Sommer P, Gutberlet M, Gaspar T, Foldyna B, Piorkowski C et al Real-time magnetic resonance-guided ablation of typical right atrial flutter using a combination of active catheter tracking and passive catheter visualisation in man: initial results from a consecutive patient series. Europace 2016;18:572–7. 26316146 10.1093/europace/euv249

[12] Nazarian S, Kolandaivelu A, Zviman MM, Meininger GR, Kato R, Susil RC et al Feasibility of real-time magnetic resonance imaging for catheter guidance in electrophysiology studies. Circulation 2008;118:223–9. 18574048 10.1161/CIRCULATIONAHA.107.742452 PMC2826501

[13] Toupin S, Bour P, Lepetit-Coiffé M, Ozenne V, Denis de Senneville B, Schneider R et al Feasibility of real-time MR thermal dose mapping for predicting radiofrequency ablation outcome in the myocardium in vivo. J Cardiovasc Magn Reson 2017;19:14. 28143574 10.1186/s12968-017-0323-0 PMC5286737

[14] Grothoff M, Gutberlet M, Hindricks G, Fleiter C, Schnackenburg B, Weiss S et al Magnetic resonance imaging guided transatrial electrophysiological studies in swine using active catheter tracking—experience with 14 cases. Eur Radiol 2017;27:1954–62. 27553931 10.1007/s00330-016-4560-7

[15] Roujol S, Ries M, Quesson B, Moonen C, Denis de Senneville B. Real-time MR-thermometry and dosimetry for interventional guidance on abdominal organs. Magn Reson Med 2010;63:1080–7. 20373409 10.1002/mrm.22309

[16] de Senneville BD, Roujol S, Jais P, Moonen CT, Herigault G, Quesson B. Feasibility of fast MR-thermometry during cardiac radiofrequency ablation. NMR Biomed 2012;25:556–62. 22553824 10.1002/nbm.1771

[17] Herzka DA, Tao S, Fink S, Kolandaivelu A, Guttman MA, Halperin H. Assessment of RF ablation lesions with T1 mapping. 20th Annual SCMR Scientific Sessions Abstract Supplement, 2017 pp. 203.

[18] Elbes D, Magat J, Govari A, Ephrath Y, Vieillot D, Beeckler C. Magnetic resonance imaging-compatible circular mapping catheter: an in vivo feasibilility and safety study. Europace 2017;19:458–64. 26896467 10.1093/europace/euw006

[19] Chubb H, Harrison JL, Weiss S, Krueger S, Koken P, Bloch LØ et al Development, preclinical validation and clinical translation of a cardiac magnetic resonance—electrophysiology system with active catheter tracking for ablation of cardiac arrhythmia. JACC: Clin Electrophysiol 2017;3:89. 29759398 10.1016/j.jacep.2016.07.005

[20] Kolandaivelu A, Zviman MM, Castro V, Lardo AC, Berger RD, Halperin HR. Noninvasive assessment of tissue heating during cardiac radiofrequency ablation using MRI thermography. Circ Arrhythm Electrophysiol 2010;3:521–9. 20657028 10.1161/CIRCEP.110.942433 PMC3410548

