# Supplementary material for: Epicardial electroanatomical mapping, radiofrequency ablation, and lesion imaging in the porcine left ventricle under real-time magnetic resonance imaging guidance—an in vivo feasibility study
Source: Europace. 2017 Dec 26;20(FI2):f254–62. doi: 10.1093/europace/eux341 (PMC6140436; doi:10.1093/europace/eux341)
Supplement: Supplementary Data [file eux341_supplementary_data.docx]

**Epicardial electroanatomical mapping, radiofrequency ablation and lesion imaging in the porcine left ventricle under real time MRI guidance – an in-vivo feasibility study**

Supplementary data:

Supplementary Figure 1 (S1): CMR-EP Set-up on a Siemens Healthcare platform (Adapted from Chubb et al. 2017, JACC: Clinical Electrophysiology with permission from Elsevier).


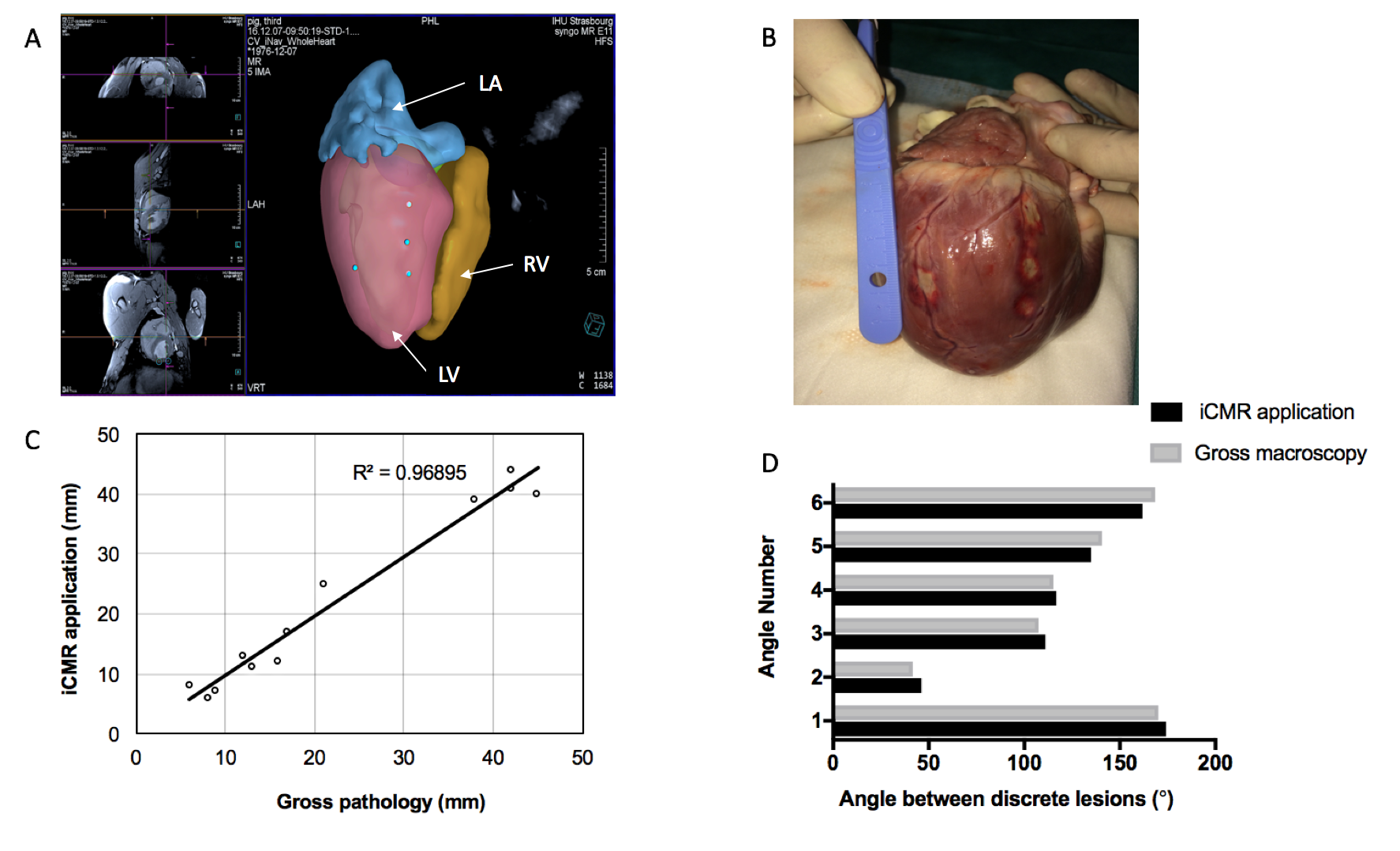


Supplementary Figure 2 (S2): Ablation lesion location on the iCMR application (Siemens Healthcare) (A) matched well to absolute site of lesion location on gross macroscopy (B). A good correlation was observed (C) with mean spatial accuracy of <3mm between the iCMR application and gross macroscopy (n=12). A conformational accuracy <7 degrees was also observed (D) in all angles measured between discrete ablation lesion lines (n=6) on the iCMR application and gross macroscopy.


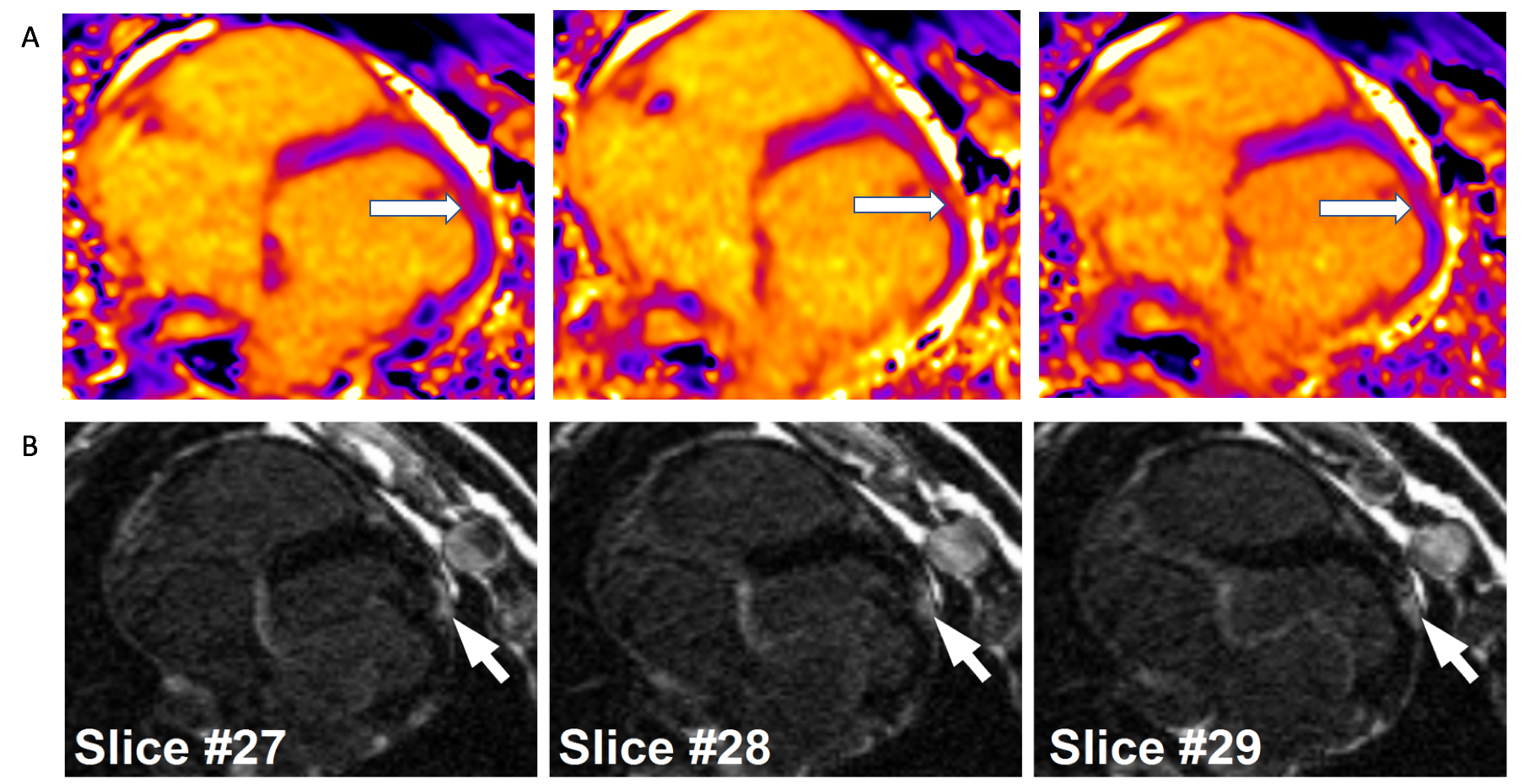


Supplementary Figure 3 (S3): Epicardial ablation areas visualized using native T1 mapping (A) matched well to ablated areas identified on late gadolinium enhancement imaging (white arrows) (B).


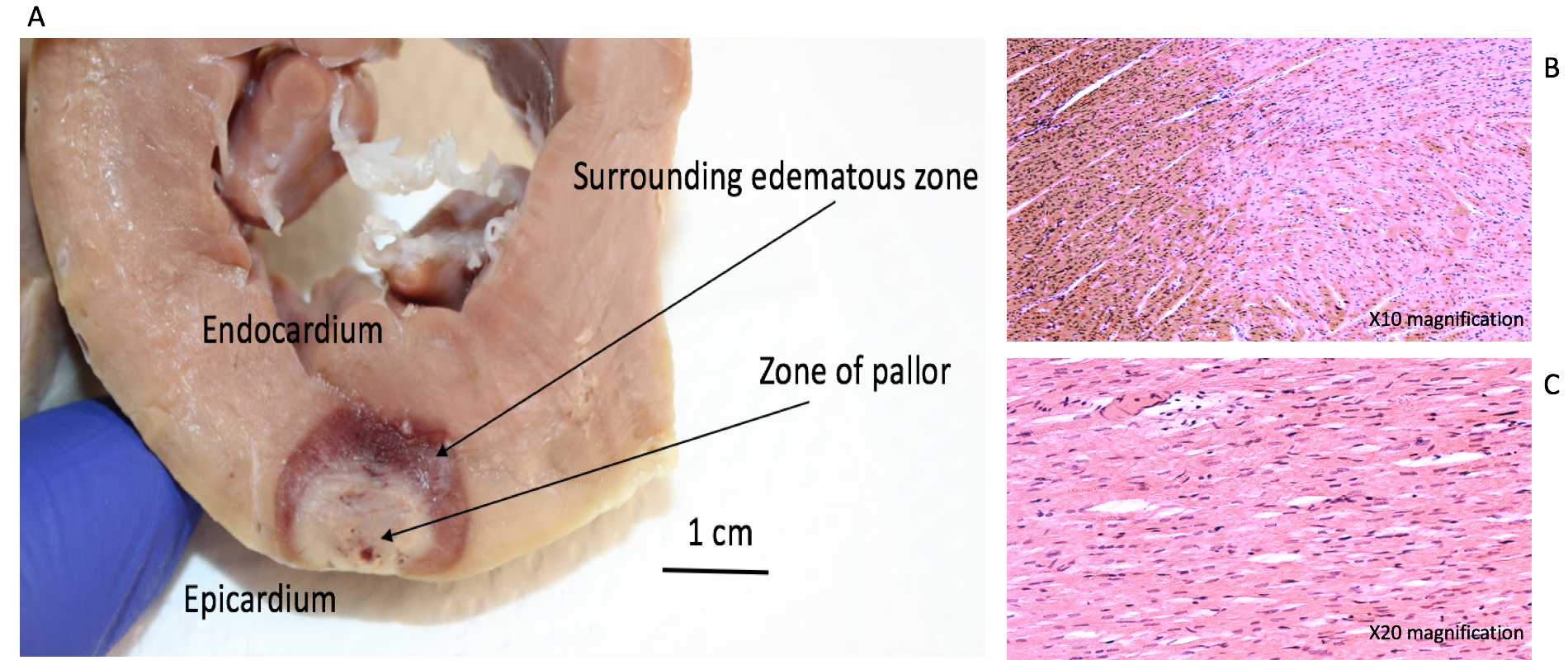


Supplementary Figure 4 (S4): A zone of pallor with a surrounding edematous zone is clearly visible on ablation lesions (A). 9/10 lesions showed a >75% transmurality. On microscopic examination (haematoxylin and eosin stain), nuclear elongation, partial loss of membrane borders and interstitial oedema was visible in ablated tissue (B and C).

Supporting text:

*Ablation strategy:* Ablation of the left ventricle was performed by delivering radiofrequency energy (40-60W, irrigation rate 17mL/min, 50-60s duration) in 4 animals. In order to assess the spatial and conformational accuracy of lesions on the iCMR application, lesions were placed in discrete locations in the left ventricle and where possible, in a ‘X’ or ‘L’ shape to enable the calculation of angles between lesions. 10 discrete ablations were delivered in 4 animals. The total number of ablations delivered in each animal was limited for the following reasons:

1. Large animal models (e.g. pigs) are particularly susceptible to arrhythmias following radiofrequency ablation in the ventricle. In preliminary work, endocardial ablations were found to be particularly likely to result in ventricular fibrillation (VF) and death of the animal. The model was less prone to arrhythmias and death following epicardial ablation, however, in order to maximize the data collected from imaging and reduce the risk of death, we elected to limit the total number of ablations in each animal.
2. In order to characterize RF delivery on CMR without bias from overlapping lesions, discrete lesions were placed in the LV with a distance of at least 1cm from two separate lesions.
